# Bacterial infection microenvironment‐responsive porous microspheres by microfluidics for promoting anti‐infective therapy

**DOI:** 10.1002/SMMD.20220012

**Published:** 2022-12-16

**Authors:** Yang Gao, Qingming Ma

**Affiliations:** ^1^ School of Pharmacy Qingdao University Qingdao China; ^2^ Key Laboratory of Functional Polymer Materials of Ministry of Education State Key Laboratory of Medicinal Chemical Biology and Institute of Polymer Chemistry College of Chemistry Nankai University Tianjin China

**Keywords:** antimicrobial therapy, bacterial infection microenvironment responsiveness, microfluidics, porous microspheres, wound healing

## Abstract

The overuse of antibiotics for treating bacterial infection has caused severe bacterial resistance and become a public health threat worldwide. It is desired to develop novel antibiotic delivery systems as efficient antibacterial strategies for promoting anti‐infective therapy. Herein, the AgNPs‐loaded *N*‐[(2‐hydroxy‐3‐trimethyl ammonium) propyl] chitosan (HTCC)/hyaluronic acid (HA) porous microspheres (HHPMs) by microfluidics have been developed as novel bacterial infection microenvironment (IME)‐responsive antibiotic delivery systems for promoting antimicrobial therapy. The release of AgNPs can respond explicitly to the IME with acidic pH values and relatively high hyaluronidase concentration. The unique porous structures of HHPMs can effectively facilitate the capture and enrichment of bacteria, thus exerting synergistic antibacterial effects, which can be more efficient in instant bacteria inhibiting and killing. The excellent biocompatibility of HHPMs is revealed by investigating their hemolytic activity and cytotoxicity. In vivo assays demonstrate that the fabricated AgNPs‐loaded HHPMs can effectively resist bacterial infection and promote wound healing and tissue regeneration at infected wound sites by inhibition of the bacterial survival. This work indicates that fabricated HHPMs are ideal bacterial infection microenvironment‐responsive materials for antibiotic delivery and show great promises for promoting anti‐infective therapy in clinics.

## Graphical abstract

Novel bacterial infection microenvironment‐response porous microspheres for delivering AgNPs have been successfully developed by microfluidics. Generated porous microspheres show responsiveness to the relatively high HAase concentration and acidic pH values in bacterial infection microenvironments to release the loaded AgNPs, and can effectively prevent bacteria from invading and infecting wound, accelerating the healing process of tissue regeneration at wound sites.

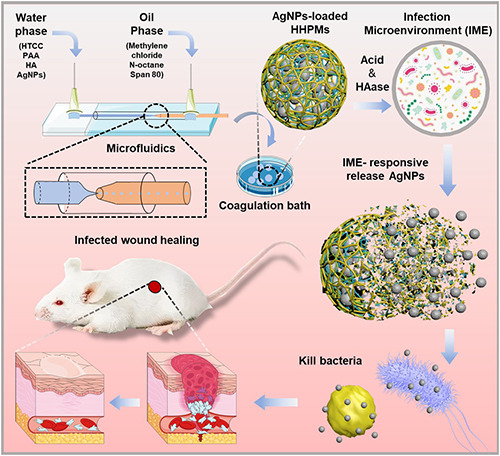

1

**Key points**

- Novel bacterial infection microenvironment‐response porous microspheres for delivering silver nanoparticles have been successfully developed by microfluidics.
- The unique porous structures can efficiently facilitate the capture and enrichment of bacteria.
- Generated porous microspheres show responsiveness to the relatively high HAase concentration and acidic pH values in bacterial infection microenvironments to release the loaded AgNPs.
- Generated porous microspheres can effectively prevent bacteria from invading and infecting wound, accelerating the healing process of tissue regeneration at wound sites.

## 2 INTRODUCTION

Bacterial infection has attracted increasing concerns as a public health threat worldwide due to its widespread transmission and prolonged treatment.^1^ As an effective antibacterial strategy, the antibiotics have been developed and studied for decades, ever since Fleming discovered penicillin in 1929.^2^ However, the overuse of antibiotics has caused severe bacterial resistance, resulting in potential contemporary infection treatment that threatens to be a future medical disaster.^3^ Thus, more efficient strategies to deliver antibiotics with optimized therapeutic effects and minimised side effects are imminent and requisite for promoting anti‐infective therapy.

Over the decades, various delivery systems have demonstrated their potentials in the delivery of antibiotics, including biological hydrogel,^4^ composite fiber,^5^ and micro/nanoscale particles.^6^ For instance, MXene‐integrated hydrogel microneedle patches can effectively promote the release of adenosine under NIR irradiation, which is beneficial for angiogenesis and wound healing.^7^ Moreover, 3D‐structured slippery microfibers can be prepared by the microfluidic 3D printing technology for medical drainage around wounds.^8^ The special surface porous structure can efficiently accelerate the exudation drainage and reduce tissue injury. Based on this feature, it is demonstrated that the textile coupled with a vacuum sealing drainage therapy could significantly enhance the wound exudation drainage efficiency, reduce tissue injury, and prolong the effective service life in versatile wound management. Thus, it is believed that the slippery microfiber textiles have potential for clinical applications.

Among all the developed systems in existence, porous microspheres have caught extensive attention with their ability to achieve high bacterial capture and enrichment.^9^ Specifically, the porous structure can increase the specific surface area to promote the exudate adsorption, which is beneficial to capture and enrich bacteria when applied at the infected site.^10^ Consequently, the relative concentration of bacteria around/inside the microspheres is increased, improving the contact and interaction of antibiotics with bacteria and resulting in enhanced antibacterial effects. Apart from the capture and enrichment of bacteria, the targeted delivery of antibiotics at the infected site is also desired for developing antibiotic delivery systems.^11^ The unique properties of the bacterial infection microenvironment (IME) offer great inspirations and possibilities for the bacteria‐targeting delivery of antibiotics.^12^ Specifically, the pH value of the bacterial infected sites decreases slightly, creating a special acidic microenvironment with pH ranges 5.5–6.5.^13^ Moreover, bacteria can release hyaluronidase (HAase) to facilitate bacterial invasion for infection, creating a relatively high HAase concentration in the IME.^12^ ^a^ Therefore, bacterial infection microenvironment‐responsive porous microspheres with pH and HAase responsiveness can be designed and exploited for targeting delivery of antibiotics to achieve efficient antimicrobial therapy.

Herein, we construct the *N*‐[(2‐hydroxy‐3‐trimethyl ammonium) propyl] chitosan (HTCC)/hyaluronic acid (HA) porous microspheres (HHPMs) as smart delivery systems for bacterial infection microenvironment‐responsive release of antibiotics to promote anti‐infective therapy. Microfluidics is used to facilitate the fabrication process with high controllability and repeatability.^14^ Silver nanoparticles (AgNPs) are synthesized and used as the representative antibiotic.^15^ HTCC is a kind of quaternised chitosan derivatives that can offer enhanced antibacterial activity while inheriting the excellent biocompatibility.^16^ Moreover, as the major constituent of the extracellular matrix, HA possesses unique beneficial functions, such as biosafety and biodegradability.^17^ HTCC and HA can be self‐assembled to form a pH‐responsive copolymeric matrix of the microspheres by the electrostatic interaction between amino groups of HTCC with carboxyl groups of HA.^18^ Besides, the fabricated HHPMs can also be hydrolyzed by the relatively high HAase concentration in the IME. Thus, HHPMs can specifically release AgNPs, responding to the bacterial infection microenvironment. In addition, HHPMs can also efficiently capture and enrich bacteria with the assistance of its porous structure and the electropositivity of HTCC.^19^ The AgNPs‐loaded HHPMs exert synergistic antibacterial effects, which can be more efficient in instant bacteria inhibiting and killing. In vitro and in vivo experiments demonstrate that the fabricated AgNPs‐loaded HHPMs can be applied as effective antibacterial materials to promote the bacterial‐infected wound healing. These features indicate that the fabricated HHPMs are ideal antibacterial materials with bacterial infection microenvironment responsiveness for antibiotic delivery and exhibit enormous values for promoting anti‐infective therapy in clinics.

## 3 RESULTS AND DISCUSSION

### 3.1 Characterization of AgNPs and HTCC

**FIGURE 1:**
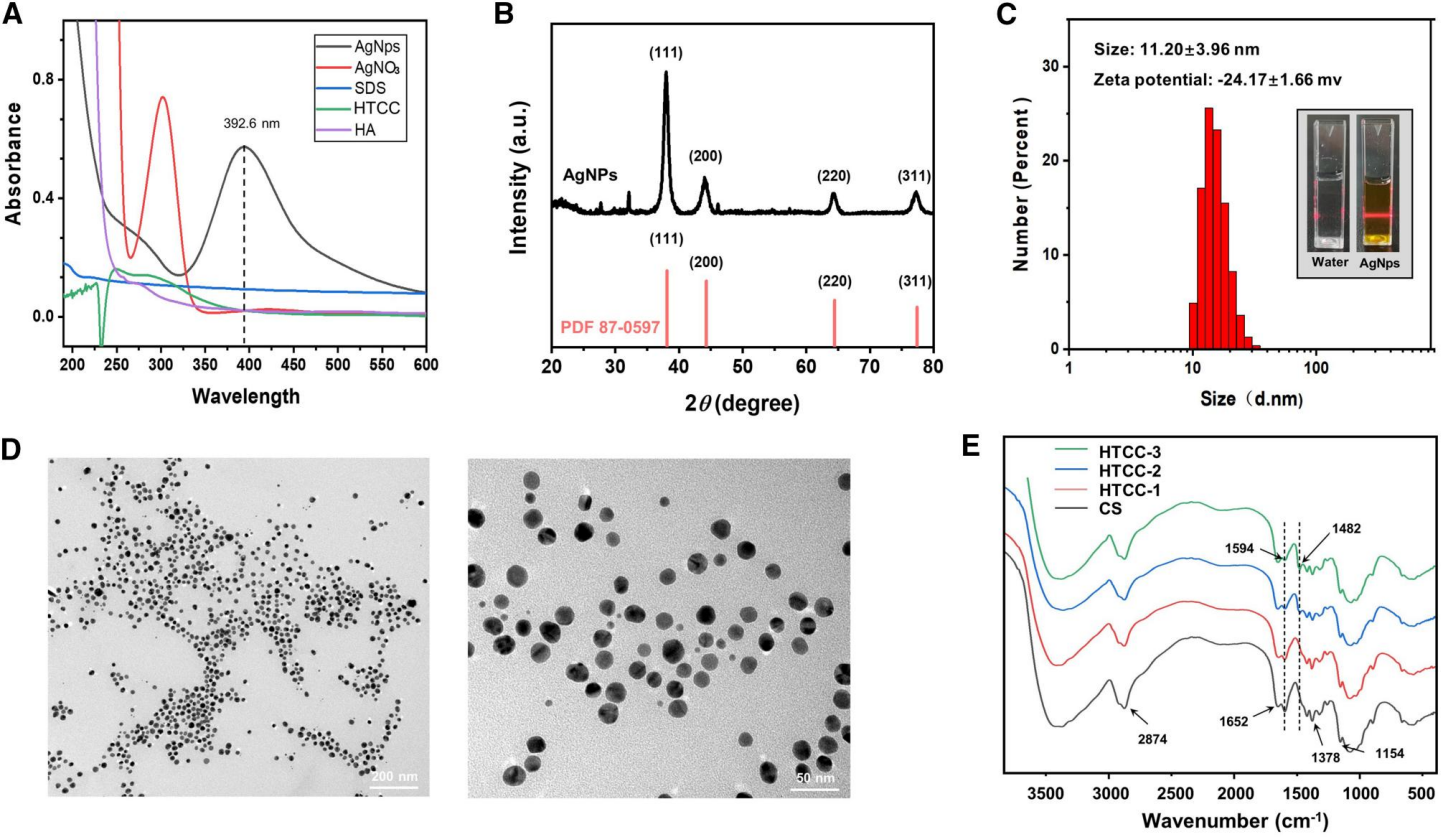
Synthesis and characterization of AgNPs and HTCC. (A) UV–vis absorption spectra of AgNPs and other components used. (B) XRD pattern of AgNPs and the standard control information. (C) The diameter distribution and Tyndall effect of AgNPs. (D) TEM images of AgNPs. (E) FTIR spectra of CS and synthesized HTCC. JCPDS is the abbreviation of Joint Committee on Powder Diffraction Standards.

The AgNPs are synthesized by the liquid phase chemical reduction method with sodium borohydride. In the synthetic process, the silver ions are reduced into silver seed clusters, which finally become the Ag nanoparticles as the reduction reaction progress. Moreover, the addition of SDS can improve the stability and monodispersity of AgNPs as previously reported.^20^ The synthesized AgNPs are first investigated by UV‐Vis spectroscopy. As shown by the spectra of UV‐Vis absorption in Figure 1A, the maximum absorption peak of the synthesized AgNPs is 392.6 nm, which undergoes a distinct red shift compared with that of AgNO_3_ solution. Moreover, the resultant characteristic absorption peak of 392.6 nm for the synthesized AgNPs also corresponds to that of solid silver nanoparticles (400 nm), which is due to the surface plasmon resonance (SPR) phenomenon.^21^ What's more, powder X‐ray diffraction (XRD) is used to further study the crystal structure of AgNPs.^22^ As demonstrated by the XRD pattern in Figure 1B, there are four intense diffraction peak signals at 2*θ* = 38.11°, 44.29°, 77.39°, and 64.44°, which refer to the (111), (200), (220), and (311) crystallographic planes, respectively. The results are anastomotic with standard cards of crystalline silver with a face‐centered cubic structure (JCPDS NO. 87‐0597). Moreover, dynamic light scattering (DLS) results reveal that the hydrodynamic diameter of AgNPs is 11.20 ± 3.96 nm, and obvious light scattering (Tyndall effect) can verify the nanoscale structure (Figure 1C). Zeta potential analysis shows that zeta‐potential of AgNPs is −24.17 ± 1.66 mv. Besides, the morphology of AgNPs is directly investigated by TEM. As shown in Figure 1D, the synthesized AgNPs sizing around 10 nm are homogeneous and dispersive.

The quaternization of chitosan to synthesize HTCC is a substitution reaction on the amino group as illustrated schematically in Figure S1 in the Supporting Information. Three ratios between CS and GTA (1.0:0.5, 1.0:1.0, and 1:1.5, respectively) are chosen to synthesize different HTCC, termed as HTCC‐1, HTCC‐2, and HTCC‐3, respectively. Infrared spectroscopy is employed to verify the synthesis of HTCC as shown in Figure 1E. Compared with the infrared spectrum of chitosan, the new C–H bending vibration peak of methyl (–CH_3_) on HTCC appears at 1482 cm^−1^. What's more, the bending vibration peak of N–H (1594 cm^−1^) gradually weakens with the increase of GTA dose. These results demonstrate that the hydroxypropyltrimethyl ammonium chloride has been added to the amino group of chitosan, and HTCC can be successfully synthesized. Furthermore, we find out that the antibacterial effect of HTCC over *Staphylococcus aureus* and *Escherichia coli* can also be increased when increasing the degree of quaternization as shown in Figure S2a. However, HHPMs prepared by excessive quaternised chitosan (HTCC‐3) are unstable and cracked as shown by the SEM images in Figure S2b. Therefore, HTCC‐2 is selected for the subsequent fabrication and application of HHPMs.

### 3.2 Microfluidic fabrication and characterization of HHPMs

**FIGURE 2:**
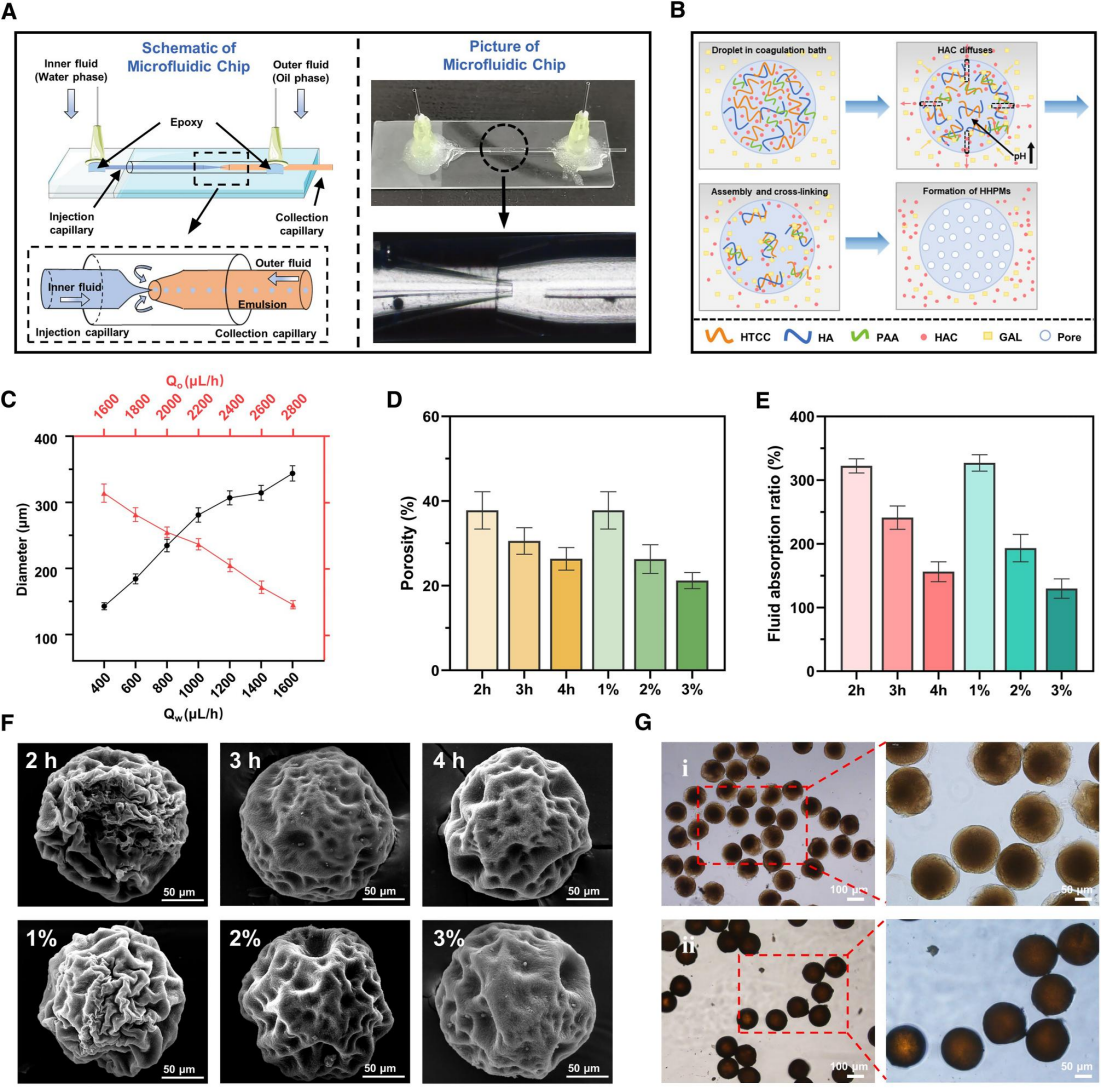
Microfluidic construction and characterization of HHPMs. (A) Schematics of the microfluidic preparation of HHPMs. (B) The proposed mechanism of the formation and coagulation of HHPMs. (C) The influence of the flow rate of both phases on the size of prepared HHPMs. (D) Measured porosity of HHPMs with different degrees of cross‐linking. The HHPMs are coagulated at 1%–3% GLA for 2 h and coagulated at 1% GLA for 2–4 h, respectively. (E) Fluid absorption ratio with different degrees of cross‐linking. (F) SEM images of HHPMs with different degrees of cross‐linking. The HHPMs are prepared as mentioned above. (G) Optical micrographs of HHPMs (i) before and (ii) after freeze‐drying

The microfluidic chip with coaxial capillary glass tubes is used to fabricate the HHPMs as shown in Figure 2A. The formation of HHPMs can be divided into four stages as illustrated schematically in Figure 2B. First, due to the stable flow rate difference between the two phases, the water phase (an aqueous solution of HTCC, PAA, HA, and acetic acid) is sheared into uniform droplets by the oil phase (a mixture of methylene chloride and n‐octane) at the tip of the capillary. The generated droplets are finally collected in the coagulation bath (oil phase with Span 80 and GLA). Then, HAC will diffuse out from the droplets due to the concentration gradient in the coagulation bath, leading to the rise of pH value from the interior to the surface of the droplet, as illustrated by the schematics in Figure 2B. The increased pH value will further induce the recovery of the electrostatic interactions between amino and carboxyl groups. Therefore, the self‐assembly between the HTCC (positively charged) with the HA and PAA (negatively charged) can be triggered to generate the basic framework of the HHPMs. Simultaneously, the framework can be crosslinked by the Schiff base reaction between the aldehyde group of GLA in the coagulation bath and the amino group of HTCC. Moreover, the diffusion of HAC will result in creating multiple flow channels, which lead to the formation of various pores in the HHPMS during the assembly and crosslinking stage.^23^ Besides, the matrix of the HHPMs will shrink during freeze‐drying due to the loss of huge amounts of water, resulting in the enlargement of the pores and the formation of porous structures of the fabricated HHPMs.

The morphology and size of HHPMs can be well controlled by tuning various factors in the fabrication process. As shown by the plots in Figure 2C, the particle size of HHPMs can be regulated by manipulating the flow rate of both phases. Particularly, when the flow rate of water phase gradually increases, the volume of the water phase passing through the tip of the capillary also enhances in unit time, resulting in the formation of larger droplets. Moreover, the high rate of water flow causes a relative reduction of the shear force generated by the oil phase. On the contrary, with the increase of the flow rate in the oil phase, the shear force toward the water phase is improved to produce smaller droplets, corresponding to the decrease of microsphere size. Since the smaller and uniform size is more beneficial for microspheres to accomplish the intimate interaction with the disease site and the stable release of drugs, 400 mL/h of water phase flow and 2800 mL/h of oil phase flow are selected to prepare HHPMs in the following experiments. The average size of optimal HHPMs is 146.24 ± 6.27 μm. Furthermore, the porous morphology of HHPMs is closely related to the coagulation time and the amount of GLA as shown by the SEM images in Figure 2F. When increasing the coagulation time from 2 to 4 h, the HHPMs' surface becomes less wrinkled with fewer pores. Same trend can also be observed when increasing the amount of GLA from 1% to 3%. Moreover, software Image J is used to measure the porosity of the cross section of HHPMs (Figure 2D). Specifically, the porosity of the HPPMs is decreased from 37.81 ± 4.42% to 26.37 ± 2.68% with increasing the coagulation duration from 2 to 4 h, which is similar to the result of increasing GLA concentration from 1% to 3% (porosity from 37.81 ± 4.42% to 21.22 ± 2.68%). Further, when excess GLA and coagulation time are implemented (such as 4% GLA with 8 h coagulation), the HHPMs turn compact and smooth with no porosity as shown in Figure S3. This phenomenon can be attributed to the densification of HHPMs caused by the enhanced degree of crosslinking. Particularly, after the amino groups of the HTCC polymer chain are complexed with carboxyl groups, the extra amino groups can be cross‐linked by GLA to further promote the formation and densification of microspheres. With the increase of coagulation time and GLA, HHPMs will be completely solidified into compact solid spheres without porosity. Moreover, the inferior porosity is adverse to the fluid adsorption, thus impeding the capture and enrichment of bacteria in the anti‐infection application of HHPMs. As shown in Figure 2E, compared with HHPMs coagulated at 1% GLA concentration for 2 h, the fluid adsorption ratio of HHPMs at 3% GLA for 2 h can be drastically reduced from 322.54 ± 11.05% to 129.85 ± 15.12%. Therefore, to obtain the excellent adsorption capacity, the HHPMs are cross‐linked and coagulated at 1% GLA for 2 h. What's more, the optical micrographs of the optimized HHPMs are shown in Figure 2G. As can be observed, the surface of HHPMs before freeze‐drying is rough and irregular. After freeze‐drying, the HHPMs show more compact structures due to the water loss, resulting in the decrease of the size form 146.24 ± 6.27 to 124.4 ± 3.24 μm. This porous structure of HHPMs can be applied in resist bacterial infection, such as adsorbing exudate and increasing local bacterial concentration to promote therapeutic effects.

### 3.3 Bacterial infection microenvironment‐responsive release properties of HHPMs

**FIGURE 3:**
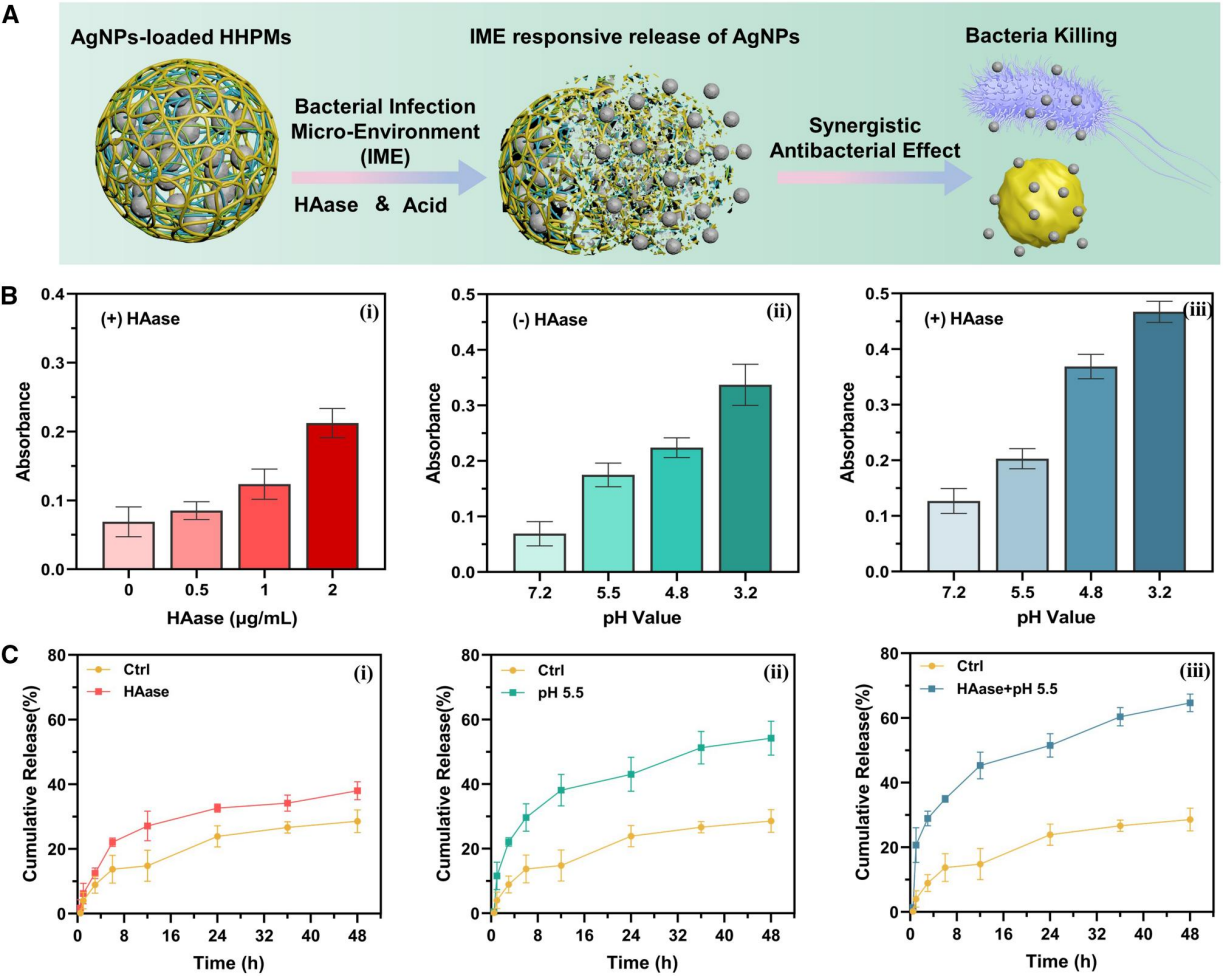
The IME‐responsive release of AgNPs‐loaded HHPMs. (A) Schematic diagram of the IME‐responsive release of HHPMs under acidic pH values and HAase. (B) UV–vis absorbance of released AgNPs in different release mediums, including various HAase concentrations (0, 0.5, 1, and 2 ug/mL, respectively) and pH values (pH 3.2, 4.8, 5.5, and 7.2, respectively). (C) Cumulative release of AgNPs from HHPMs in different release mediums at different pH values with or without HAase, respectively.

The AgNPs as antibacterial are added into the water phase to prepare the AgNPs‐loaded HPPMs. Due to the unique characteristics of microfluidics such as precisely manipulate tiny fluids and create stable liquid interface in microchannels,^15^ the resultant HPPMs can exhibit acceptable drug encapsulation capacity even at high AgNPs concentrations. The encapsulation efficiency of AgNPs in HHPMs is 67.66 ± 4.40%, and the loading efficiency is 0.98 ± 0.08%. Furthermore, the prepared HHPMs show distinct bacterial infection microenvironment responsiveness and can release the encapsulated AgNPs when under acid pH values or subjected to HAase as illustrated in Figure 3A. Particularly, the HA in the HHPMs will gradually degrade when contact with HAase, leading to the breakup of the polymeric matrix, thus resulting in accelerating the release of the AgNPs from the unstable HHPMs. As shown in Figure 3B(i), the absorbance of the release medium for AgNPs‐loaded HPPMs increases from 0.069 ± 0.022 to 0.212 ± 0.021 when increasing the amount of HAase in the release medium from 0 µg/mL to 2 µg/mL. Moreover, as demonstrated by the plots in Figure 3C(i), the existence of HAase in the release medium can help accelerate the release of AgNPs. Particularly, the cumulative release within 48 h can be increased from 28.95 ± 3.49% to 38.01 ± 2.82% when 1 µg/mL HAase is added in the release medium. Besides, since acidic conditions would deteriorate the cross‐link between HTCC and GLA, the fabricated HHPMs also show pH responsiveness. When the pH value is decreased from 7.2 to 3.2, the absorbance of the release medium for AgNPs‐loaded HPPMs increases from 0.069 ± 0.022 to 0.337 ± 0.037 as shown in Figure 3B(ii). Further, the cumulative release within 48h can be increased from 28.95 ± 3.49% to 54.22 ± 5.27% when the pH value of the release medium is changed from neutral to acidic (pH 5.5) (Figure 3C(ii)). Further, when the acidic release medium contains HAase, the release capacity of HHPMs is fully activated, resulting in the maximum of AgNPs release as demonstrated in Figure 3B(iii),C(iii). These results indicate that the release capacity can be influenced by HAase and pH, revealing the bacterial infection microenvironment responsiveness of HHPMs.

### 3.4 In vitro antibacterial activity

**FIGURE 4:**
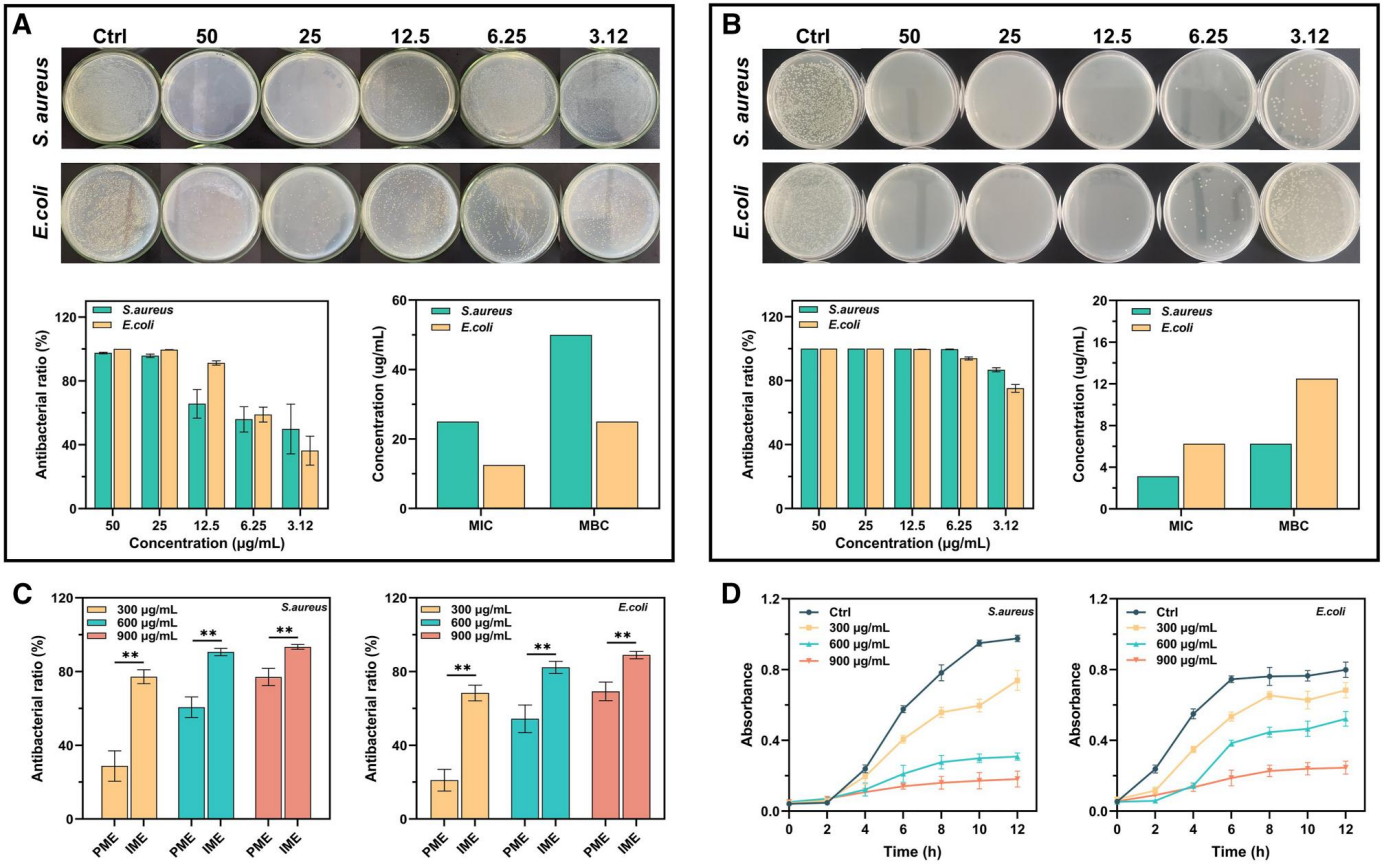
The antibacterial effect of the AgNPs and AgNPs‐loaded HHPMs against *Staphylococcus aureus* and *Escherichia coli*. The results of MIC and MBC analyzes for (A) AgNPs and (B) AgNPs‐loaded HHPMs. The concentration mentioned in panel (B) represents AgNPs relative concentration converted by drug loading efficiency from HHPMs. (C) Antibacterial ratio of 300, 600, and 900 μg/mL AgNPs‐loaded HHPMs in physiological microenvironment (PME) and infection microenvironment (IME). (**p < 0.01). (D) Inhibitory effects of AgNPs‐loaded HHPMs on the growth of *S. aureus* and *E. coli* in LB liquid media.

To evaluate the antibacterial properties of the synthesized AgNPs and fabricated AgNPs‐loaded HHPMs, their MIC and MBC are determined at different concentrations. As shown in Figure 4A,B, both AgNPs and AgNPs‐loaded HHPMs exhibit dose‐dependent antibacterial effects. The MIC and MBC of AgNPs against *S. aureus* are 25 and 50 µg/mL, respectively, while that for *E. coli* is 12.5 and 25 µg/mL. What's more, AgNPs loaded in HHPMs are more efficient in antagonizing *S. aureus* compared to *E. coli*. The concentration mentioned in Figure 4B is the relative concentration of AgNPs loaded in microspheres, which represents the content of AgNPs‐loaded HHPMs to compare the antibacterial activity. Moreover, the MIC and MBC of AgNPs loaded in HHPMs against *S. aureus* are 3.12 and 6.25 µg/mL, lower than the results obtained from *E. coli* (6.25 and 12.5 µg/mL), indicating that AgNPs‐loaded HHPMs are more efficient in antagonizing *S. aureus* compared to *E. coli*. After loading AgNPs into HHPMs, the enhanced antibacterial effect can be attributed to the synergetic inhibitory action of HTCC. Besides, the bacterial infection microenvironment (IME) is artificially constructed by preparing acetic acid buffer solution (pH 5.5) contained 1 ug/mL HAase. The results of colony forming units are immune from the interference of IME, compared with the physiological microenvironment (PME) simulated by PBS of pH 7.2 (Figure S4). The antibacterial ratio of AgNPs‐loaded HHPMs can be further improved in bacterial infection microenvironment (IME, acetic acid buffer of pH 5.5 contained 1ug/mL HAase) as indicated in Figure 4C. Specifically, with the increasing AgNPs‐loaded HHPMs concentration from 300 μg/mL to 900 μg/mL in the PME, the antibacterial ratios increase from 28.80 ± 8.24% to 77.07 ± 4.66% for *S. aureus* and 21.08% ± 5.84% to 69.26 ± 5.04% for *E. coli*, respectively. Besides, since AgNPs can be responsively released from HHPMs by the acid and HAase, the antibacterial ratios of 600 μg/mL HHPMs are 90.62 ± 2.01% for *S. aureus* and 82.29 ± 3.29% for *E. coli* in the IME and are observably higher than that of 60.65 ± 5.57% for *S. aureus* and 54.36 ± 7.51% for *E. coli* in the PME (*p* < 0.01). Similar results are consistent with the antibacterial ratios of 300 and 900 μg/mL of AgNPs‐loaded HHPMs. This result confirmed the bacterial infection microenvironment‐responsive release of the fabricated AgNPs‐loaded HHPMs. For further research to the inhibitory effects of AgNPs‐loaded HHPMs on bacterial growth, the dose‐dependent growth kinetics curves are detected for *E. coli* and *S. aureus* treated by AgNPs‐loaded HHPMs at different concentrations (Figure 4D). AgNPs‐loaded HHPMs can slow and even inhibit the growth of *S. aureus* and *E. coli*. What's more, the inhibitory effect over *S. aureus* is better than that of *E. coli*. This phenomenon may be attributed to the production of HAase by *S. aureus*, which can accelerate the release of AgNPs from the HHPMs.^24^ Since the *S. aureus* cell wall is composed by peptidoglycan and is more susceptible to reactive species, HTCC can destroy the cell wall and biofilm to promote the antibacterial effect of AgNPs. On the contrary, the outer cell wall of *E. coli* is composed of lipopolysaccharides, lipoproteins, and phospholipids, which can defend against the attack of reactive species moderately.^25^

**FIGURE 5:**
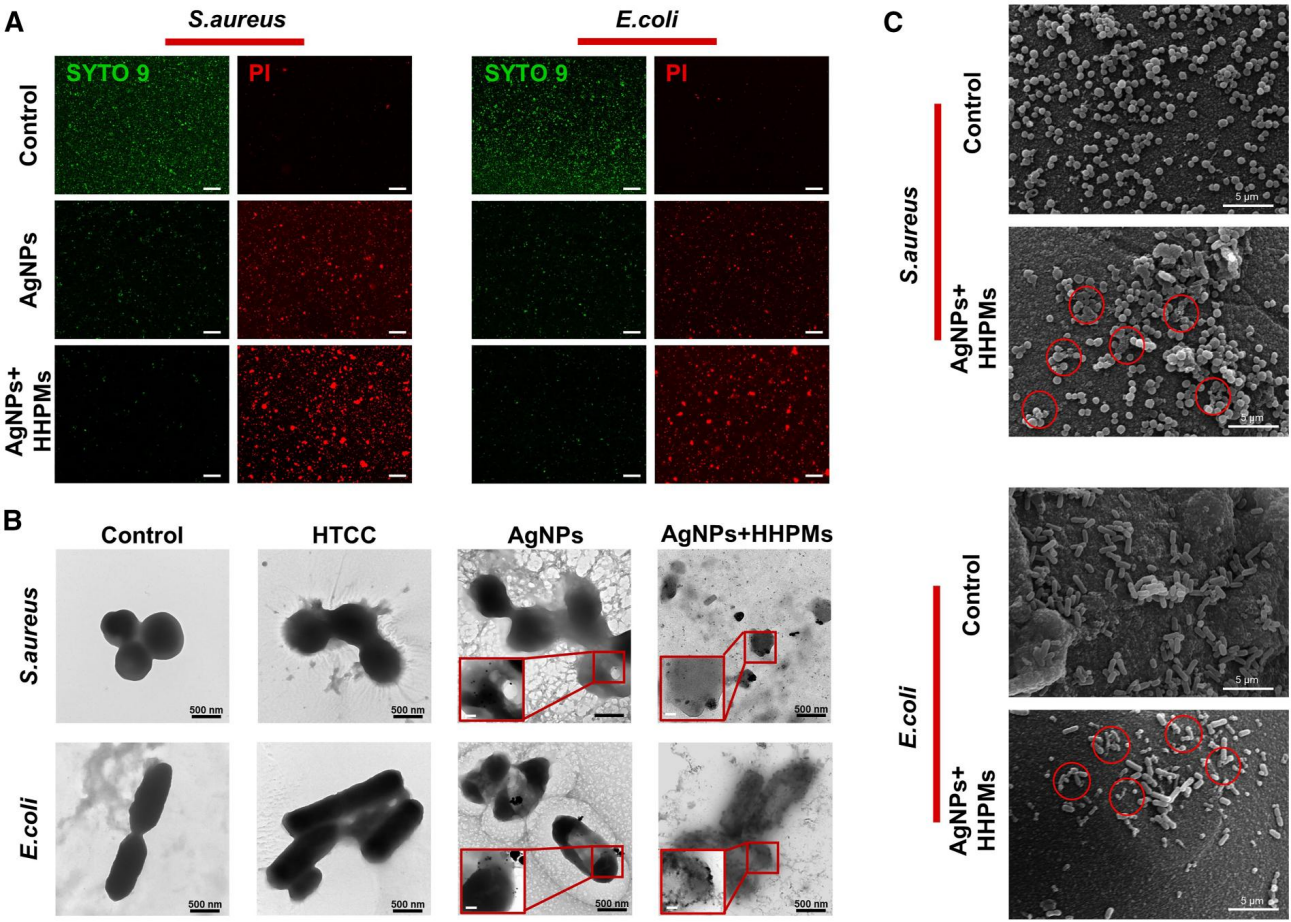
Observing state and micromorphology of bacteria before and after treated by different samples. (A) Images of bacterial live/dead fluorescent staining. Scale bar is 50 μm. (B) SEM images of bacteria untreated and treated by HHPMs for 2 h. (C) TEM images of bacteria untreated and treated by HTCC (50 μg/mL), AgNPs (6 μg/mL), and preprocessed HHPMs (600 μg/mL AgNPs‐loaded HHPMs incubated with IME for 12 h). Scale bar of the inset is 200 nm.

Bacterial live/dead fluorescent staining in in vitro antibacterial studies is conducted to further demonstrate the effective antibacterial capacity of AgNPs‐loaded HHPMs. As shown by the fluorescence images in Figure 5A, strong green signals can be detected in the control group, revealing the viable state of the bacteria. After treating with tested samples for 24 h, more red signals can be observed in the AgNPs‐loaded HHPMs group than in the AgNPs group, demonstrating the stronger effective antibacterial ability of the fabricated AgNPs‐loaded HHPMs. What's more, the scanning electron microscopy is used to directly reveal the adsorption and destruction effects of HHPMs against the *S. aureus* and *E. coli* (Figure 5C). *S. aureus* and *E. coli* in the control group exhibit normal morphology, retaining smooth cell walls and intact profile. While after treated by AgNPs‐loaded HHPMs for 2 h, both bacteria adsorb on the surface and suffer different degrees of shrinkage and deformation (as highlighted by the red circle). Moreover, the ultrastructure of bacteria is also observed by TEM. As shown in Figure 5B, both *S. aureus* and *E. coli* in the control group are distinct with intact morphology and smooth surface. While when treated by HTCC and AgNPs, their edges become unclear, and cavities and resident AgNPs can be seen inside the bacteria, indicating enhanced bacterial destruction of HTCC and AgNPs. Moreover, in the AgNPs‐loaded HHPMs group, bacteria are severely damaged and unable to maintain their morphology, leaving blurry profiles and extensive leakage. Particularly, *S. aureus* turns into fragments without a continuous cellular structure. These microscopic observations of bacteria confirm the effective antibacterial properties of the fabricated AgNPs‐loaded HHPMs. As previously mentioned, the positive electrical properties of HTCC can damage the bacterial cell walls and impair the defense and protection of bacteria against AgNPs. In the IME, HHPMs can adsorb bacteria due to the abundant positive charges and unique porous structure, which increases the bacterial concentration around the microspheres. Meanwhile, AgNPs can be specifically released and directly contacted with bacteria to avoid being diluted and resultant reduction of its efficacy.

### 3.5 Biocompatibility

**FIGURE 6:**
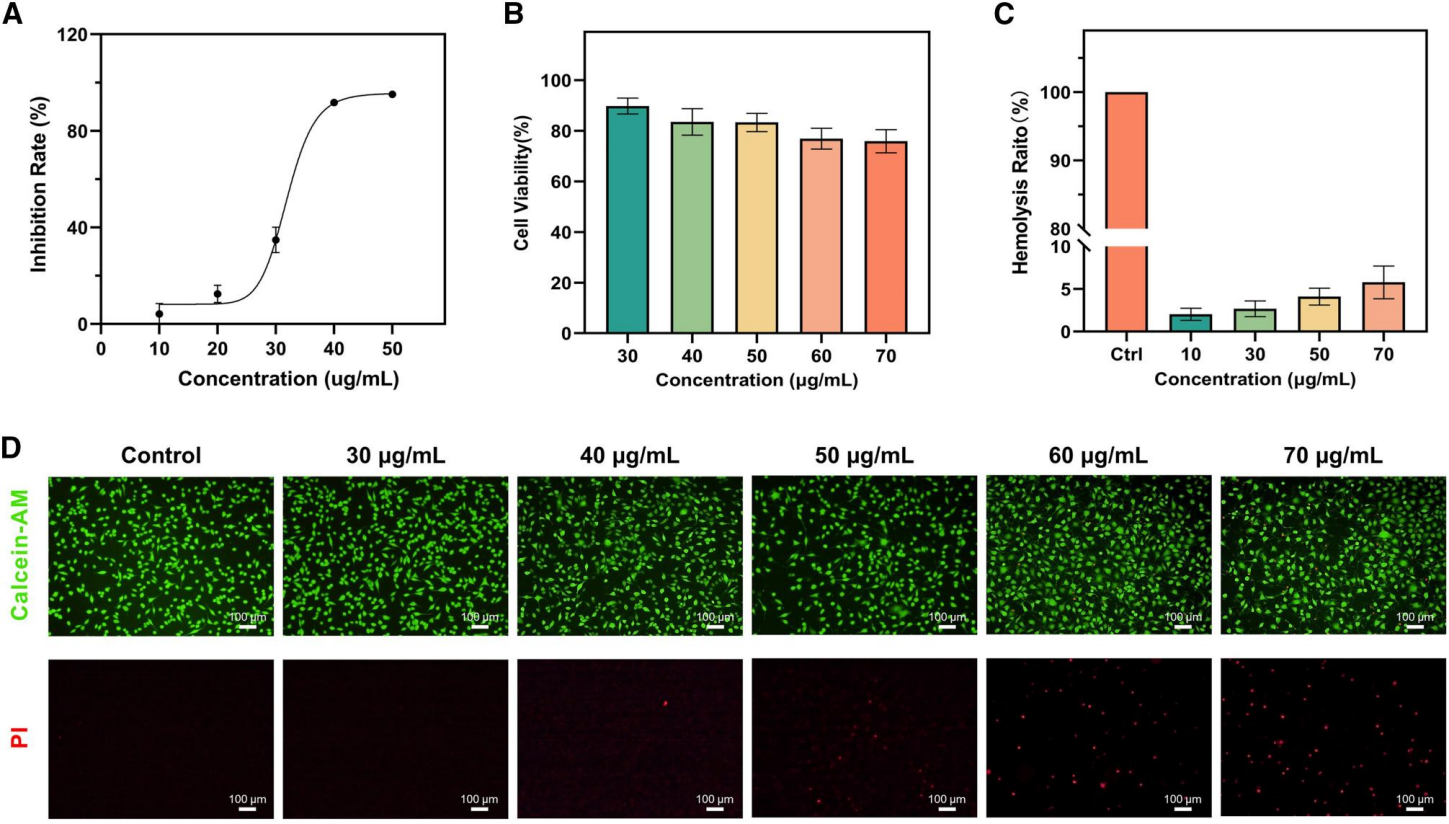
Biocompatibility of AgNPs and HHPMs. (A) The relation between inhibition rate and concentration of AgNPs. (B) Cell viability of L929 cells after treated with different concentrations of AgNPs‐loaded HHPMs. (C) Hemolysis evaluation of AgNPs‐loaded HHPMs. (D) Calcein‐AM)/(PI) staining of L929 cells treated by AgNPs‐loaded HHPMs. The concentration mentioned in panels (B–D) represents AgNPs relative concentration converted by drug loading efficiency from HHPMs.

To evaluate the biocompatibility of synthesized AgNPs and fabricated HHPMs, the cytotoxicity of L929 mouse fibroblasts is analyzed by the MTT method. As shown in the dose‐response curve in Figure 6A, the 50% inhibitory concentration of AgNPs is 31.95 ± 0.43 μg/mL. Moreover, when the concentration of AgNPs reaches about 24 μg/mL, the cell viability can reach 90%. It is worth noting that biosafety of AgNPs can be greatly improved when encapsulated into HHPMs. For instance, after encapsulated in the HHPMs, even the relative concentration of AgNPs is up to 70 μg/mL, cell viability can exceed 70%, as shown by the plot in Figure 6B. Moreover, the cell live/dead staining results also confirm the cytocompatibility of HHPMs (Figure 6D). The green signal representing live cells dominates in the fluorescent staining images. In addition, the hemolysis ratio is lower than the standard of 5% in all HHPMs groups as demonstrated in Figure 6C. Furthermore, the cell viability and hemolysis ratio for all the materials used for constructing AgNPs and HHPMs are also verified as demonstrated in Figure S5. These results illustrate the biosafety of AgNPs and HHPMs, which show the promises to be used in vivo.

### 3.6 In vivo antibacterial activity and wound healing

**FIGURE 7:**
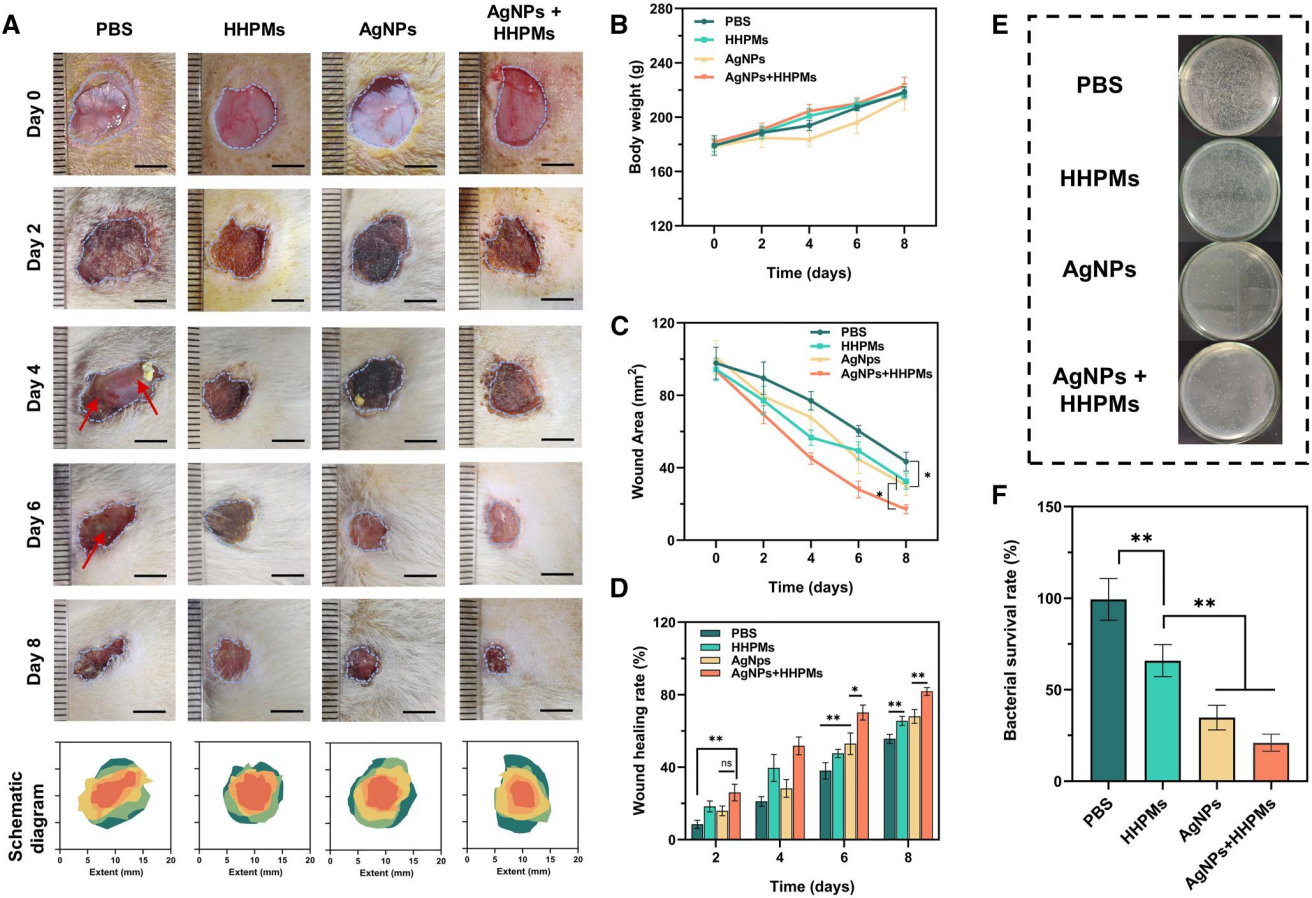
Evaluation of in vivo antibacterial ability and healing of bacterial infected wound. (A) Representative photographs and diagrams of the wound in different groups on days 0, 2, 4, 6, and 8. Scale bars: 5 mm. (B) Body weight of different groups during the process of treatments. (C) Wound area of different groups during the process of treatments. (D) Comparison of wound healing rate among different groups (*p < 0.05, **p < 0.01). (E) Bacterial colonies obtained from infected tissues treated in different groups. (F) Corresponding bacterial survival rate of infected tissues treated in different groups (*p < 0.05, **p < 0.01).

To further investigate the capacity of fabricated AgNPs‐loaded HHPMs on promoting in vivo bacterial inhibition and wound healing, we construct an infected skin wound model on the rat's dorsal site. Wounds are incubated with *S. aureus* and then treated with PBS, blank HHPMs, AgNPs, and AgNPs‐loaded HHPMs, respectively. The photos of the wounds treated with different samples are shown in Figure S6. The entire process of wound healing is monitored at different time intervals (Figure 7A). During the treatment period, the body weight of the rats remains relatively stable as demonstrated by the plot in Figure 7B. Rats treated with PBS develop severe inflammation on the day 4, indicating that residual bacterial have produced local abscesses and ulcers, as illustrated by the red arrows in Figure 7A. Compared with the control group treating by PBS, wound healing is significantly faster in the AgNPs and AgNPs‐loaded HHPMs groups as shown in Figure 7C. What's more, the wound healing can be further accelerated after treating with AgNPs‐loaded HHPMs. Specifically, as demonstrated by the schematic diagrams in Figure 7A and the plots in Figure 7D, the wound healing rate is 26.01 ± 4.64% after treating with AgNPs‐loaded HHPMs on the second day, which is significantly higher than that of 8.53 ± 2.18% for the PBS group (*p* < 0.05). Besides, the rate of wound healing of HHPMs and AgNPs on the second day is not different from that of the control group. However, in the late stage of wound healing (days 6 and 8), the disparity of wound healing rates gradually increases among the four groups, indicating the excellent facilitation of AgNPs‐loaded HHPMs in promoting wound healing. Furthermore, to verify the antibacterial effect in vivo, the bacteria are collected from wounds on the second day and incubated on LB agar plates. The results show that the number of colonies from the AgNPs and AgNPs‐loaded HHPMs groups is significantly less than the other groups (*p* < 0.01) as demonstrated by the images of the LB agar plates with different treatments in Figure 7E. Moreover, the bacterial survival rate declines drastically from 99.37 ± 11.36% in the PBS group to 25.06 ± 4.69% in the AgNPs‐loaded HHPMs group (Figure 7F). These results validate the superior in vivo antibacterial efficacy of AgNPs‐loaded HHPMs, which can effectively facilitate bacterial infected wound healing.

## 4 CONCLUSION

In summary, we successfully developed a novel bacterial infection microenvironment‐response HHPMs for loading and releasing AgNPs by applying microfluidic technology. The unique porous structures of HHPMS can efficiently facilitate the capture and enrichment of bacteria. Moreover, HHPMs can be triggered to release the loaded AgNPs in bacterial infection microenvironments with relatively high HAase concentration and acidic pH values. Moreover, dissociative HTCC can also destroy the bacterial wall, which is conducive to support an intracellular antibacterial mechanism of AgNPs. Therefore, the fabricated AgNPs‐loaded HHPMs exert synergistic antibacterial effects, which can be more efficient in instant bacteria inhibiting and killing. Since the components used for constructing AgNPs and HHPMs possess high biocompatibility, HHPMs exhibit favorable biosafety in the evaluation of cell viability and hemolysis ratio. Furthermore, in vivo experiments demonstrate that the resultant AgNPs‐loaded HHPMs can quickly and effectively prevent the bacteria from invading and infecting wound, accelerating the healing process of tissue regeneration at wound sites. These results indicate the developed HHPMs could be a promising candidate for promoting anti‐infective therapy in clinics.

## 5 EXPERIMENTAL SECTION

### 5.1 Materials

Chitosan (CS, 85% deacetylation degree) was supplied by Yuanye Bio‐Technology (Shanghai, China). The aqueous solution of 25.0 wt.% poly acrylic acid was bought in J&K Scientific (Beijing Bailingwei Biological Technology Co., Ltd). Hyaluronic acid (HA, Mw 10000) and glycidyl trimethyl ammonium chloride (GTA) were supplied by Aladdin (Shanghai, China). Acetic acid buffers were brought from Phygene (Fuzhou Feijing Biological Technology Co., Ltd). Phosphate‐buffered saline (PBS, pH 7.2), and methyl thiazolyl tetrazolium (MTT) were supplied by Sigma‐Aldrich. The 25 wt.% poly (acrylic acid) aqueous solution, hyaluronidase (HAase), silver nitrate (AgNO_3_), and glutaraldehyde (GLA) were supplied by Macklin (Shanghai, China). Calcein‐AM/PI living/dead cell double staining kits were bought from Solarbio (Beijing, China). Live/dead bacterial staining kit was supplied by Shanghai Gaochuang Chemical Technology Co., Ltd. Dulbecco's Modified Eagle Media (DMEM), fetal bovine serum (FBS), Trypsin‐EDTA, and penicillin/streptomycin were supplied by Gibco (USA).

### 5.2 Synthesis and characterization of AgNPs

The AgNPs in this study was synthesized by the NaBH_4_ reduction method. Specifically, the aqueous solution of AgNO_3_ (120 μL, 0.1 M) and SDS (0.12 g) was mixed in water (30 mL) and kept stirring at room temperature. After the complete dissolution of SDS, 5 mL of NaBH_4_ aqueous solution (0.5 mg mL^−1^) was cautiously and slowly dripped in. Stir (3 h at room temperature) was applied, then the product was dialyzed (MWCO 500) against ultrapure water with a frequent exchange for 72 days to remove the needless substance. The synthesized AgNPs was centrifuged (11,962×*g*, 15 min) and dried for further research. UV‐Vis spectra of AgNPs were analyzed by UV–vis spectroscopy (UV‐2600; Shimadzu, Japan). The X‐ray diffraction was obtained by the X‐ray diffractometer (BRUKER D8 Advance, USA) and used for phase identification and crystal structure analysis. The transmission electron microscopy (TEM, JEM‐1200E, JEOL) was used to characterize the morphology of AgNPs, and the dynamic light scattering (DLS, Zetasizer Nano ZS, Malvern) was used to evaluate the size distribution and polydispersity index.

### 5.3 Synthesis and characterization of HTCC

Firstly, 2 g of chitosan was dissolved in an aqueous solution containing 2% (v/v) acetic acid. 1 M NaOH aqueous solution was added to the above solution until the pH reached to 8–9 to prepare alkalized chitosan. Then, the corresponding alkalized chitosan was added to 30 mL acetone containing certain amounts of GTA at 80°C. After 8 h of constant temperature reaction, the precipitated products were washed with acetone and dried by vacuum to get HTCC. The synthesized HTCC was characterized by FTIR analyses.

### 5.4 Microfluidic fabrication of HTCC/HA porous microspheres (HHPMs)

The microfluidic device used was fabricated with coaxial capillary glass tubes. A glass capillary (inner diameter 1.2 mm) was chosen as the outer tube. Two capillary glass tubes with an outer diameter of 1.0 mm were selected as the inner tubes. Among the two inner capillary tubes, the tip diameter of one tube was polished to 80 μm and used as the injection tube, and the other was polished to 500 μm and used as the collection tube. Moreover, two microsyringe pumps were used to adjust and regulate the flow rates of the dispersed and mobile phases, respectively. Herein, the water phase was an aqueous solution containing 1.5 wt.% HTCC, 0.1 wt.% PAA, 0.3 wt.% HA, and 40% (v/v) acetic acid, respectively. The oil phase was a mixture of methylene chloride and n‐octane (concoct = 7:3, v/v), containing 2.0 wt.% Span 80 as the surfactant. The coagulation bath was the oil phase containing additional glutaraldehyde (GLA) as a cross‐linker. After coagulation (two to four h, room temperature), n‐octane was used to wash the prepared HTCC/HA porous microspheres three times and dried by a freeze‐drier method for 24 h.

### 5.5 Fluid adsorption ratio of HHPMs

After drying at 60°C for 1 h in a vacuum oven, the weight of dried HHPMs was measured. Then, the dried samples were put into a centrifuge tube contained distilled water, and the centrifuge tube was put into a 37°C incubator for 30 min. Before measuring the weight of wet samples, the centrifuge tubes were centrifuged at 8000 rpm for 15 min to remove excess water. The weight of wet and dried samples was recorded as M_1_ and M_0_, respectively. The following equation was used to calculate the fluid absorption ratio:

(1) $$Fluid\;absorption\;ratio\;=\;\left(M_{1}-M_{0}\right)/{\text{M}}_{0}\times 100\text{\%}.$$

### 5.6 HAase and pH‐responsive release of AgNPs from HHPMs

To investigate the triggered release of AgNPs from HHPMs under HAase and acidic pH values, the entrapment efficiency of AgNPs was firstly measured by detecting the concentration of AgNPs in supernatant. The AgNPs were added into the water phase to prepare AgNPs‐loaded HPPMs. After collection and coagulation of HHPMs, the supernatant of the coagulation bath was separated. The supernatant absorbance of the unencapsulated AgNPs was measured at the maximum absorption wavelength by the UV–vis spectrophotometer. After calculating the encapsulation and loading efficiency, the release behavior of AgNPs was tested in different mediums. Briefly, 40 mg HHPMs were immersed in a 10 mL release medium. At different time intervals, 1.0 mL of release medium was fetched, followed by addition of the same volume of fresh medium. The concentration of AgNPs was determined by an ultraviolet spectrophotometry. In this work, different release mediums contained acetate buffer, HAase, and a combination of acetate buffer and HAase, respectively. In addition, all mediums contained 0.1 wt.% L‐ascorbic acid to prevent the oxidation of AgNPs. All samples were prepared and measured in triplicate.

### 5.7 Antibacterial assays

#### 5.7.1 Minimal inhibition and bactericidal concentration (MIC and MBC) assays

*Staphylococcus aureus* (*S. aureus*, ATCC 29213) and *Escherichia coli* (*E. coli*, ATCC 8739) were selected as the representative gram‐positive and gram‐negative microorganisms to measure MIC and MBC of samples. Briefly, the bacteria were grown in the LB medium under shaking gently (100 rpm) for 24 h at 37°C. Then, the obtained bacterial suspensions were diluted to 1 × 10^6^ CFU/mL. Different doses of samples were added to an equal volume of bacterial suspensions, resulting in final concentrations of AgNPs of 0, 3.12, 6.25, 12.5, 25, and 50 µg/mL, respectively. After coculturing for 24 h at 37°C, 100 μL of bacterial suspension was fetched and spread across the surface of the solid agarose medium evenly. After incubation at 37°C for 18 h, the colony units on the solid agarose were investigated and recorded. Based on the results of the antibacterial ratio, the lowest sample concentration that can inhibit 90% and 99% of bacterial growth was used as the MIC and MBC, respectively. The following equation was used to calculate the antibacterial ratio:

(2) Antibacterial Ratio (%) = (Nc−Ns)/Nc×100%,

where Ns and Nc were the bacterial colony numbers in the sample and the control, respectively.

#### 5.7.2 Bacterial growth inhibition assay in culture

The culture of *E. coli* and *S. aureus* was diluted to approximately 1 × 10^6^ CFU/mL by detecting the optical density (OD600 = 0.05). Afterward, 20 mL of obtained bacterial culture were cocultured, respectively, with HHPMs at different concentrations. The bacterial suspension was incubated with continuous agitation (100 rpm) at 37°C, and the bacterial concentrations were monitored every 2 h through the measurement of OD600. Each experiment was repeated in triplicate.

#### 5.7.3 Live/dead staining of bacteria assays

The live/dead staining of bacteria was applied to illustrate and verify the antibacterial capacity of the samples. *E. coli* and *S. aureus* suspensions (100 μL, 10^6^ CFU/mL) were removed and cocultured with the samples at 37°C. Live/Dead Bacterial Viability Kit (L‐13152, Thermo‐Fisher Scientific, Shanghai, China) was used to stain the bacterial suspension for 15 min. After staining, the samples were photographed with the confocal laser scanning microscopy (CLSM, LSM510, Zeiss).

#### 5.7.4 Bacteria morphology observation

Bacterial suspensions were cocultured with the samples at 37°C for 2 h, followed by the addition of 2.5% glutaraldehyde. After centrifugal washing, gradient ethanol solutions (30%–100%, v/v) were used to dehydrate the samples sequentially with each concentration for 10 min. The dried sample was vacuumed and then investigated by the scanning electron microscope (SEM, Type S‐4800, Hitachi). Moreover, after centrifugal washing, the bacterial suspensions fixated by glutaraldehyde were characterized by TEM.

### 5.8 Cytocompatibility assays

In vitro cytocompatibility of samples was investigated by the MTT assay using L929 mouse fibroblast cells. 100 μL of L929 cells were seeded in 96‐well microplates (1 × 10^4^ cells per well) and cultured for 24 h in DMEM (5% CO_2_, 37°C). Then, the cells were respectively treated with different samples for further incubating 48 h. Cells without treated by samples were used as the control.

After removing the original medium, 100 μL MTT solution (5 mg mL^−1^) was added to each well for incubation with 4 h. Finally, reacted MTT solutions were removed, and formazan crystals were dissolved by the addition of the DMSO. The absorbance for each well was tested at 590 nm with the microplate reader (Thermo Scientific MK3). Each experiment was repeated in triplicate. The following equation was used to calculate the cell viability:

(3) Cell viability (%) = Sample/Control×100%.

Living/dead cells fluorescence staining was also performed to estimate the cytocompatibility of the samples. L929 cells were firstly inoculated in a 24‐well plate (2 × 10^4^ cells per well) with the samples at 37°C for 24 h, before staining by the propidium iodide (PI, red 11 fluorescence) and Calcein‐AM (Calcein‐AM, green fluorescence) for 15 min. Cell staining status was observed using an Olympus IX‐53 fluorescence microscope.

### 5.9 Hemocompatibility

The hemolysis ratio was measured to evaluate the in vitro hemocompatibility of the samples. Specifically, 500 mL of 2.0% fresh mouse erythrocyte suspension was added to the equal amount of sample suspension and incubated for 3 h (37°C). The compound was then centrifuged for 10 min with 3000 rpm. The microplate reader was used to measure the absorbance of supernatant at 560 nm. Equal volumed distilled water and saline solution were served as the positive and negative control, respectively. Each experiment was repeated in quintuplicate. The following equation was used to calculate the hemolysis ratio:

(4) Hemolysis (%) = (S−N/P−N)×100%,

where P, N, and S were the absorbance of the positive control, negative control, and sample, respectively.

### 5.10 In vivo antibacterial capacity and healing study on the infected wound

The animals used in the experiment were treated according to the Qingdao University Laboratory Animal Welfare Ethics Committee Approval (No. 20200602SD352012064155).

After general anesthetization, the SD rats were shaved to clean epidermal hair on the back. A biopsy sampler (10 mm diameter) was used to make a dorsal trauma model of SD rats. Full‐thickness wound on dorsal skin was made by a biopsy sampler (10 mm). The infected wound model was further constructed by the addition of *S. aureus* suspension (100 μL of 1 × 10^7^ CFU/mL per rat) on the skin wound site. After invasion and attack of bacteria for 2 h, the infected sites were treated with PBS (50 μL), AgNPs (50 μL, 25 μg/mL), HHPMs (20 mg), and AgNPs‐loaded HHPMs (20 mg), respectively. Each rat was individually fed in a cage to avoid from licking or rubbing the wound. The healing of the wound was monitored as at 2, 4, 6, and 8 days after treatment, while the same determination is executed for the weight of rats. Based on the change of the wound area, the following equation was used to calculate the wound healing rate:

(5) $$Rate\;of\;wound\;healing\;\left(\text{\%}\right)\;=\;\left(A_{0}-A_{n}\right)/A_{0}\times 100\text{\%},$$

where A_0_ and A_n_ were the areas on the day 0 and day n (2, 4, 6, and 8).

Furthermore, to explore the bactericidal effect in vivo, the treated bacterial samples at the wound site before treatment were collected as the initial control group. Subsequently, the bacterial samples at the wound site treated with PBS, HHPMs, and AgNPs‐loaded HHPMs were also collected as test groups, respectively. All bacterial were added to LB agar plates and counted colonies to calculate the bacterial survival rate.

(6) $$Bacterial\;survival\;rate\;\left(\text{\%}\right)\;={\text{ N}}_{t}/N_{c}\times 100\text{\%},$$

where N_t_ and N_c_ were the colony numbers in the test and control groups, respectively.

## AUTHOR CONTRIBUTIONS

Yang Gao: Conceptualization; Data curation; Formal analysis; Visualization; Writing – original draft. Qingming Ma: Resources; Funding acquisition; Conceptualization; Supervision; Writing – review & editing; Project administration.

## CONFLICT OF INTEREST

The authors declare no conflict of interest.

## ETHICS STATEMENT

All animal experiments were performed in accordance with the guidelines for the management and use of laboratory animals in the Qingdao University, and were approved by the Animal Ethics Committee of Qingdao University, Ethics Approval Number: 20200602SD352022063158, Jan 11, 2022.

## Supporting information

Supplementary Material
