# Supplementary material for: Bacterial infection microenvironment‐responsive porous microspheres by microfluidics for promoting anti‐infective therapy
Source: Smart Med. 2022 Dec 16;1(1):e20220012. doi: 10.1002/SMMD.20220012 (PMC11236009; doi:10.1002/SMMD.20220012)
Supplement: Supplementary file 1 — Supplementary Material [file SMMD-1-e20220012-s001.docx]

Supporting Information

Bacterial Infection Microenvironment-Responsive Porous Microspheres by Microfluidics for Promoting Anti-Infective Therapy

Yang Gao^a, b^ , Qingming Ma^a,^*

^a^ *School of Pharmacy, Qingdao University, Qingdao 266071, China*

^b^ *Key Laboratory of Functional Polymer Materials of Ministry of Education, State Key Laboratory of Medicinal Chemical Biology and Institute of Polymer Chemistry, College of Chemistry, Nankai University, Tianjin 300071, China*

*** Corresponding author.

E-mail address: qma@qdu.edu.cn (Q. Ma).

Corresponding author at: School of Pharmacy, Qingdao University, Qingdao 266071, China.


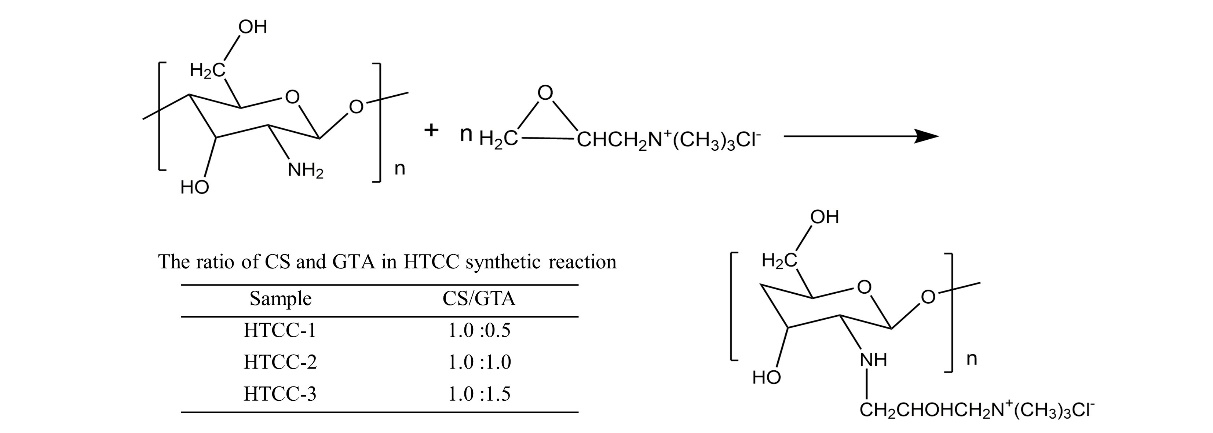


**Fig. S1.** Chemical synthesizing process of HTCC by different ratios between CS and GTA.


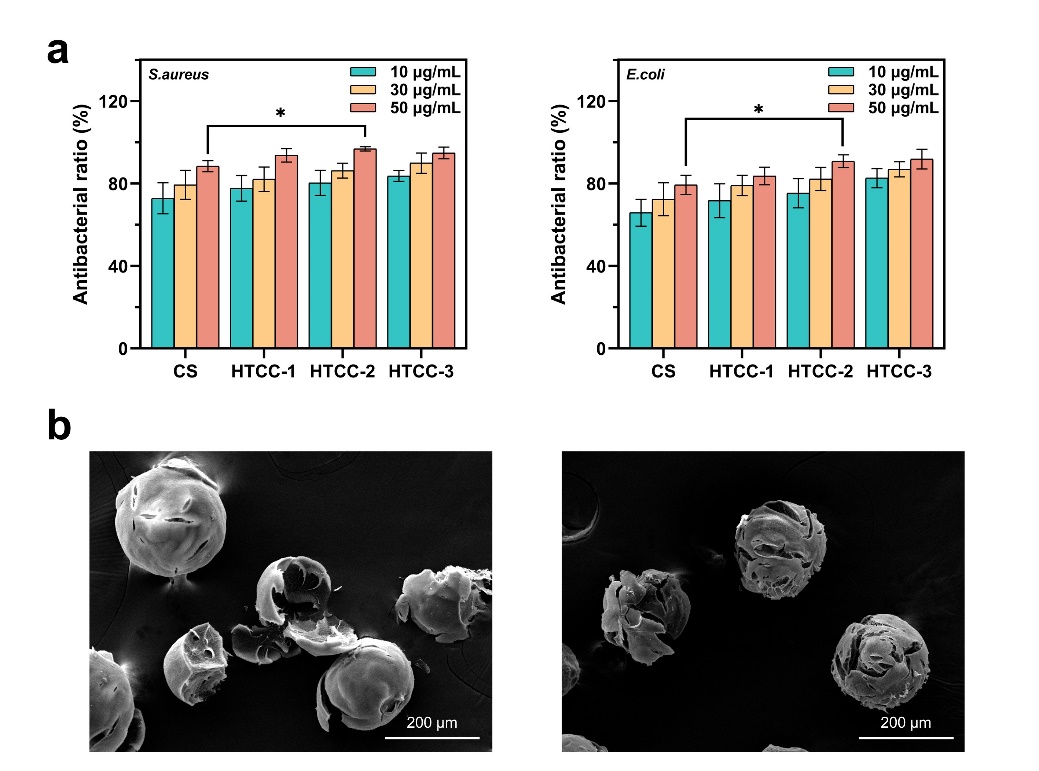


**Fig. S2.** (a) Antibacterial ratio of various HTCC at 10, 30, and 50 μg/mL. (n=3, * *P*<0.05). (b) SEM image of HHPMs prepared by HTCC-3.


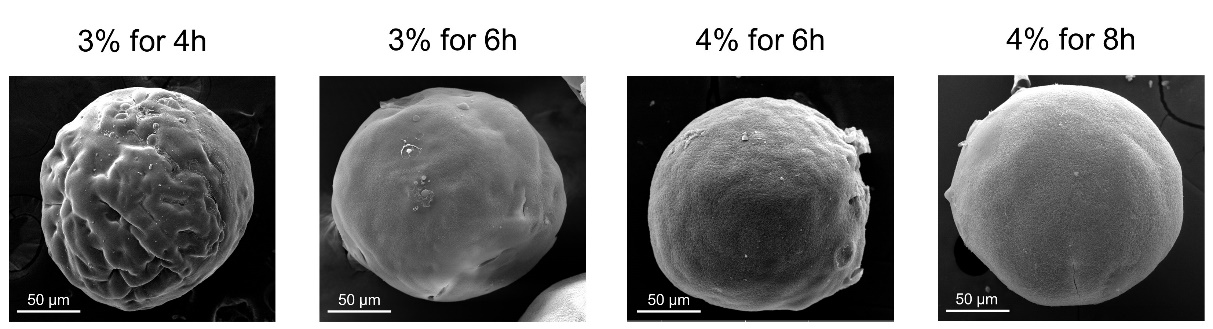


**Fig. S3.** (a) SEM images of HHPMs with excessive crosslinking.


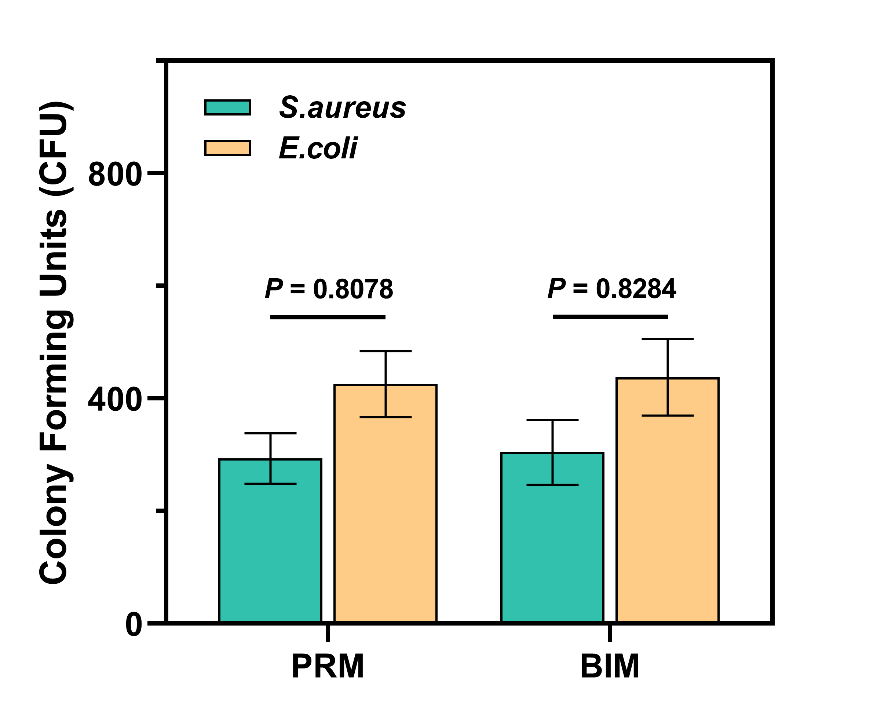


**Fig. S4.** Colony forming units of *S. aureus* and *E. coli* in in physiological microenvironment (PME) and bacterial infection microenvironment (IME).


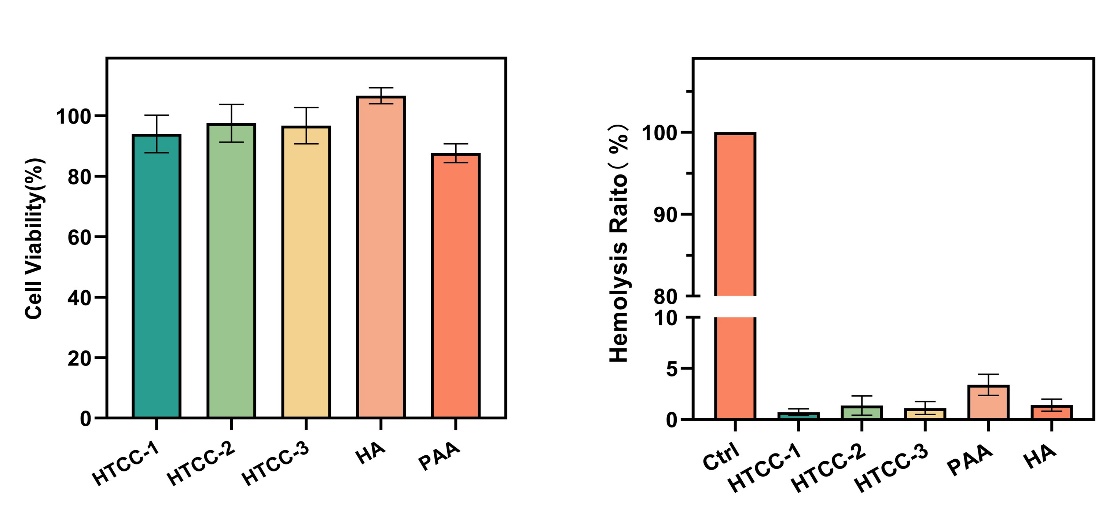


**Fig. S5.** Cell viability and hemolysis ratio of all materials used for constructing AgNPs and HHPMs.


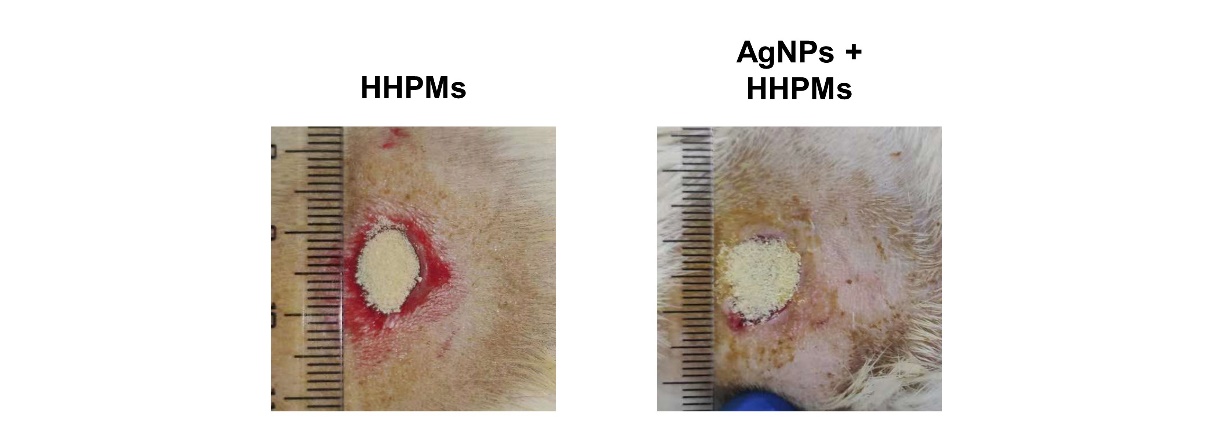


**Fig. S6.** The photos of the wounds treated with HHPMs and AgNPs-loaded HHPMs.

**The degradation of the fabricated AgNPs-loaded HHPMs *in vitro*.**

To investigate the degradation of the fabricated AgNPs-loaded HHPMs, the enzymatic degradation method is used to obtain the degradation behavior *in vitro*. Specifically, the dried AgNPs-loaded HHPMs were weighted as W_0_, and then incubated in the simulative physiological microenvironment (PME, PBS containing 10KU/mL lysozyme, pH=7.4, 37 °C), and infection microenvironment (IME, Acetate buffer containing 10KU/mL lysozyme and 1ug/mL HAase, pH=5.5, 37 °C), respectively. At different time intervals, the residue of AgNPs-loaded HHPMs was isolated by centrifugation, and then lyophilized and weighed as W_1_. The degradation ratio can be calculated by the following equation:

Degradation ratio (%) = (W_0_ – W_1_)/ W_0_ ×100 %

The results show that the degradation ratio of AgNPs-loaded HHPMs is 10.42±0.60% in the PME and 14.18±0.61% in the IME for 7 days (as shown in the Figure below), which can help explain the special IME-responsive release of AgNPs-loaded HHPMs. Moreover, we have further fitted the obtained data in to different kinetic models (zero order model, first order model, and Higuchi model respectively). The results show that the R^2^ in the zero order model is the largest among the different models for both PME (R^2^=0.9868 for zero order model, 0.977 for first order model and 0.970 for Higuchi model) and IME (R^2^= 0.9949 for zero order model, 0.9921 for first order model and 0.9723 for Higuchi model), indicating that zero order model is the best fit of the degradation kinetics of AgNPs-loaded HHPMs in the duration of action (7 days). And the degradation kinetics of AgNPs-loaded HHPMs in the duration of action (7 days) can be expressed by Y= 0.016X- 0.0095 in PME and Y= 0.021X- 0.0021 in IME, where Y represents the degradation ratio and X represents the time, respectively.


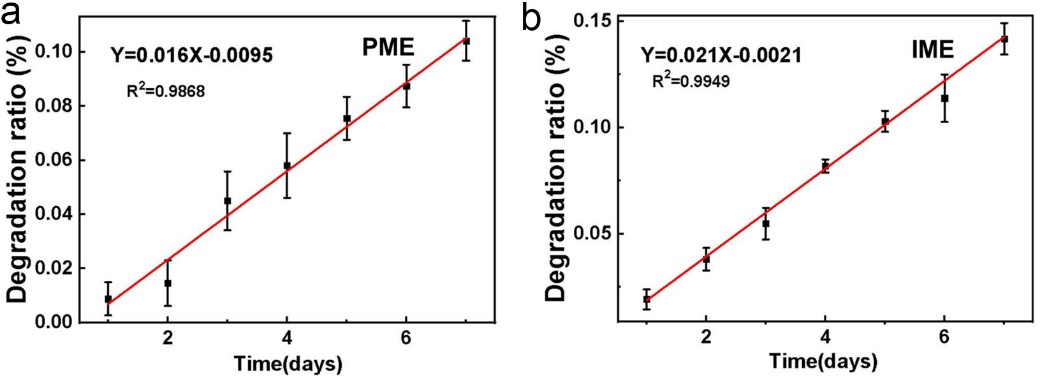


**Fig. S7**. *In vitro* degradation of AgNPs-loaded HHPMs in (a) PME and (b) IME in the duration of action (7 days). The dry samples (W_0_) were incubated in the simulative physiological microenvironment (PBS containing 10KU/mL lysozyme, pH=7.4, 37 °C), and infection microenvironment (Acetate buffer containing 10KU/mL lysozyme and 1ug/mL HAase, pH=5.5, 37 °C). At different time intervals, the residue (W_1_) was centrifuged to isolate before lyophilizing and weighting.
